# A regionally refined quarter-degree global atmospheric rivers database based on ERA5

**DOI:** 10.1038/s41597-024-03258-4

**Published:** 2024-05-03

**Authors:** Bin Guan, Duane E. Waliser

**Affiliations:** 1 Joint Institute for Regional Earth System Science and Engineering, University of California, Los Angeles, Los Angeles, California USA; 2 Jet Propulsion Laboratory, California Institute of Technology, Pasadena, California USA

**Keywords:** Atmospheric dynamics, Hydrology

## Abstract

Atmospheric rivers (ARs) are narrow, elongated, synoptic jets of water vapor that play important roles in the global water cycle. The continually developing Tracking Atmospheric Rivers Globally as Elongated Targets (tARget) algorithm identifies AR objects at individual time steps based on thresholding integrated water vapor transport (IVT) and other requirements, and tracks each AR object in time and space. Building on previous versions of tARget, this paper discusses further refinements to the algorithm to better handle ARs in tropical and polar areas, as well as “zonal” ARs which the previous versions of the algorithm were not designed to capture. This further regionally refined algorithm is applied to the ERA5 reanalysis over 1940–2023 at 6 h intervals and a 0.25° × 0.25° horizontal resolution. The AR detection results are evaluated in terms of key AR characteristics. We anticipate this regionally refined global AR database will aid further understanding of ARs such as AR process studies, evaluation of AR simulations and predictions, and assessment of climate change impacts on ARs.

## Background & Summary

### Influence of atmospheric rivers

Based on a brief record of atmospheric analysis data, the term “atmospheric river” (AR) was coined by Zhu and Newell^1^ to represent the relatively narrow filaments of horizontal water vapor transport that often occur within and across the midlatitudes. Their initial findings showed that while ARs on average only occupy about 10% of the Earth surface in the midlatitudes, they account for over 90% of the poleward transport of water vapor across those latitudes^2^. These characteristics indicate a critical role for ARs in shaping the global water and energy cycles, including their extremes.

Based on nearly three decades of research since the introduction of the concept, ARs have now been recognized as major precipitation deliverers, flood producers, and drought busters in a number of regions, and have been the focus of a rapidly increasing volume of studies in the recent few years^3,4^. For example, ARs on average account for 40% of the Sierra Nevada mountain snowpack^5^ – an essential source of water for California. On the other hand, almost all flood events on California’s Russian River are associated with ARs^6^. The global study of Paltan *et al*.^7^ highlights the role of ARs in water availability and floods in many parts of the world. The combination of beneficial and hazardous aspects of ARs underlies the introduction of the AR scale^8^ that aims to better characterize and communicate their impacts analogous to hurricane categories.

Numerous studies have put efforts into observing and understanding the characteristics and impacts of ARs near and at their landfall locations. Other studies have started to focus on the genesis and development of ARs on a global scale. Due to the lack of *in situ* observations, especially over regions away from AR landfalls, and the lack of information on vertical wind profiles from satellite observations, AR observational studies have largely relied on reanalysis products. These products routinely provide information on the vertical distribution of moisture and wind, from which a key AR variable is calculated – the vertically integrated water vapor transport (IVT). Because reanalysis products rely on a numerical weather prediction model to assimilate available observations, they are subject to errors and uncertainties of the underlying model. Evaluating the reanalysis-based results against direct observations, as done in this study, helps to understand these errors and uncertainties.

### AR detection methods

Since the introduction of the rudimentary AR detection method by Zhu and Newell^2^ and especially during the recent five years or so, over 30 AR detection methods – largely complementary to one another and intended for different applications – have been developed in the community, many of which have been evaluated and intercompared under the Atmospheric River Tracking Method Intercomparison Project^9–12^ (ARTMIP). These methods vary in the geophysical variables used, and the requirements and thresholds applied to isolate ARs from background conditions. These methods can be grouped into three broad categories: (1) methods based on a single point, such as at AR landfall locations, to best take advantage of available *in situ* observation at those locations^13,14^; (2) methods based on a pre-determined cross-section, such as along coastal segments where ARs frequently make landfall^15–17^; and (3) methods based on all grid points of a region or the globe, to consider the full footprint and movement of each AR^18–20^.

Among the third type of methods, a widely used one is Tracking Atmospheric Rivers Globally as Elongated Targets (tARget). To facilitate a global perspective, the tARget algorithm adopts a broad definition for ARs, which does not pre-exclude any regions from AR detection. Similar “permissive” approaches have also been adopted in other algorithms for global or near-global studies of ARs (e.g., those intercompared in Lora *et al*.^21^). Since its introduction, the tARget algorithm has been continually developed and improved, with a major enhancement to enable the tracking of AR life cycles in the last version – version 3^22–24^. The AR database based on this algorithm has been evaluated in a number of studies using dropsonde and satellite observations in terms of basic AR characteristics such as AR width, total IVT across the AR width, and the number of landfall dates, and has often been used as a benchmark in other studies^25–27^. It has been noted that AR characteristics may be sensitive to the AR detection method, including the resolution of the input data^9–12,28^. The tARget algorithm is found to be particularly robust to the input data (Figs. 3, 4 of Collow *et al*.^12^) – a desirable attribute in the context of method-related AR detection uncertainty.

Since its inception, the tARget algorithm – via its applicability across the globe – has provided a globally uniform approach to AR observational characterizations^29–33^, model evaluations^34–37^, and future projections^38–40^, including extending the consideration of ARs to a number of less-studied areas such as tropical, polar, inland, and east-coast areas^41–45^. The algorithm has even been extended for use in characterizing extreme aerosol transports – that is, aerosol atmospheric rivers^46,47^. The algorithm and the reanalysis-based global AR database have been used in over 100 studies so far. With the further refinements to the algorithm and details about the data record described herein, and the application to a high-resolution reanalysis product, it is our hope that the tARget algorithm/database will continue to provide a useful resource for global AR science and applications, such as further understanding of AR processes, evaluation of AR simulations and predictions, and assessment of climate change impacts on ARs.

## Methods

### tARget algorithm

The tARget algorithm comprises two major components: AR identification, and AR tracking. We refer to AR identification as methods for determining whether an AR condition is present in a given location at a given time (i.e., a Eulerian perspective), and AR tracking as methods to follow an AR in time and space over its life cycle (a Lagrangian perspective). Most of the existing AR detection methods are designed for AR identification – only a limited number of them are capable of tracking AR life cycles^48–50^. This tracking capability, along with the comprehensive recording of life cycle characteristics (lifetime, travel distance, etc.), is a key feature of the tARget AR database.

A simple method to identify ARs could be the use of a sole IVT threshold (“fixed threshold”) – a constant applied universally, such as that used by Ralph *et al*.^8^ in establishing the AR scale. While the simplicity can be useful for applications where information about AR geometry is not available or not an important consideration, a method based on a sole IVT threshold may have limitations in large-scale/global applications due to different IVT climatologies across regions. A more nuanced, time- and latitude-dependent IVT threshold (“relative threshold”) was used by Zhu and Newell^2^ to account for the varying background IVT across time and latitudes. Both fixed and relative thresholds have been used in different AR identification methods. The current AR database is based on a combination of semi-fixed (hemispherically constant but time dependent) and relative (both location- and time-dependent) IVT thresholds, along with other requirements as detailed below.

Our AR identification is based on further refining the tARget algorithm, bringing it to version 4 (hereafter, tARget version 4 or simply version 4). Here, we first describe all major steps in version 4, then point out the refinements compared to the previous version. Using zonal and meridional IVT from the ERA5 reanalysis^51^ as input, the algorithm considers a combination of intensity, geometry, and direction thresholds in identifying ARs, with the following major steps:

1. IVT objects (contiguous areas of connected pixels) are extracted based on IVT magnitude exceeding the location- and month-dependent 85^th^ percentile threshold, IVT_threshold_(x,y,m), where x and y are zonal and meridional coordinates of a pixel, and m is the month index that varies between 1 and 12. For each month, the percentile is calculated based on the five months centered on that month during 1980–2019. To facilitate AR identification in cold and/or dry regions where IVT is climatologically low, an additional, location-independent IVT threshold is applied hemispherically. This hemispheric IVT threshold is derived from the area-weighted spatial 5^th^ percentile of the pixel-wise threshold, IVT_threshold_(x,y,m), for each month and each hemisphere (see detailed discussion in the next section).
2. An object is retained if the standard deviation of IVT directions across individual pixels does not exceed 67.5°. This step serves to eliminate features with extremely incoherent IVT directions closely associated with tropical cyclones.
3. An object is retained if its axis has a contiguous segment or multiple such segments totalling longer than 1000 km where each pixel has a poleward IVT component greater than 25% of the total IVT at that pixel. This step aims to remove features with entirely zonally-directed or equatorward IVT primarily found in the tropics.
4. An object is retained if it is longer than 2000 km with a length-to-width ratio greater than 2. This step retains objects that are long and narrow, which are defining characteristics of ARs. The length and length-to-width ratio are calculated as in Guan and Waliser^24^.

IVT objects failing the geometrical and directional requirements in steps 2–4 are not immediately discarded. Instead, they are subject to additional iterations of steps 1–4 where each time the pixel-wise IVT threshold in step 1 will be raised by 2.5th percentile, up until the threshold reaches the 95th percentile. The additional iterations help to retain ARs where the surrounding object determined by the IVT 85th percentile threshold is not well-elongated, but a core region inside does satisfy the AR requirements, following a similar method used in Wick *et al*.^18^ and adopted in Guan *et al*.^23,24^. Note that even though some requirements in steps 1–4 target features mainly found in specific regions, all these requirements are applied globally, that is, without arbitrary specification of the domains where they apply.

**Fig. 1:**
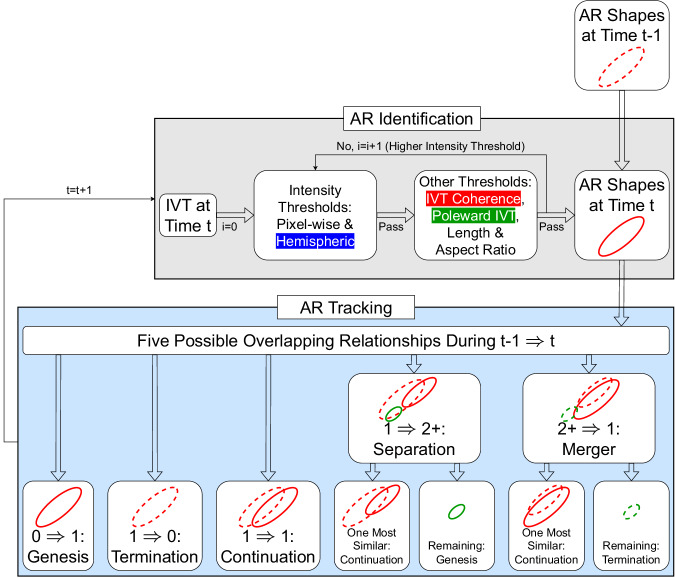
Flowchart of tARget version 4. The shaded backgrounds in light gray and light blue indicate the identification and tracking components of the algorithm, respectively. In the gray background area, white texts on a colored background indicate the refinements in version 4 (see text for details). In the blue background area, the five possible overlapping relationships between AR shapes at time steps t−1 and t are schematically shown, where each solid oval represents an AR shape at t, and the dashed ovals project AR shapes from t−1 to t. Note not all the cases have both solid and dashed ovals. For example, the genesis case only has a solid oval at t, and the termination case only has a dashed oval at t−1 (bottom left two squares). Green ovals indicate genesis as a result of separation, or termination as a result of merger. Updated from Guan and Waliser^24^.

The objects retained after steps 1–4 form the AR shapes. Once AR shapes at two adjacent time steps are obtained, a straightforward approach is used to track the movement of any given AR shape between the two time steps and construct AR life cycles. Two AR shapes, each from one of the two time steps, that spatially overlap are regarded to belong to the same life cycle. This presumes the temporal sampling interval (here, 6 h) is short enough relative to the typical AR size and travel speed such that AR shapes belonging to the same life cycle can be expected to overlap between any two adjacent time steps. In this approach, the specification of an arbitrary search radius is unnecessary as the AR shape boundary naturally serves that purpose. For a given AR life cycle, the first time step is regarded as the genesis, and the last time step the termination, and life cycle characteristics (such as lifetime and travel distance) are calculated based on that. When separation occurs, that is, when one AR shape from the previous time step overlaps with multiple AR shapes at the current time step, only one of the AR shapes at the current step that is most morphologically similar to the AR shape from the previous step is deemed continuation of the existing AR life cycle, and the remaining AR shapes at the current time step are each deemed genesis of a new life cycle. The mirroring case, AR merger, is handled in an opposite manner. A summary of the AR identification and tracking steps described above for tARget version 4 is shown in Fig. 1.

### Refinements in version 4

#### Tropical refinement

**Fig. 2:**
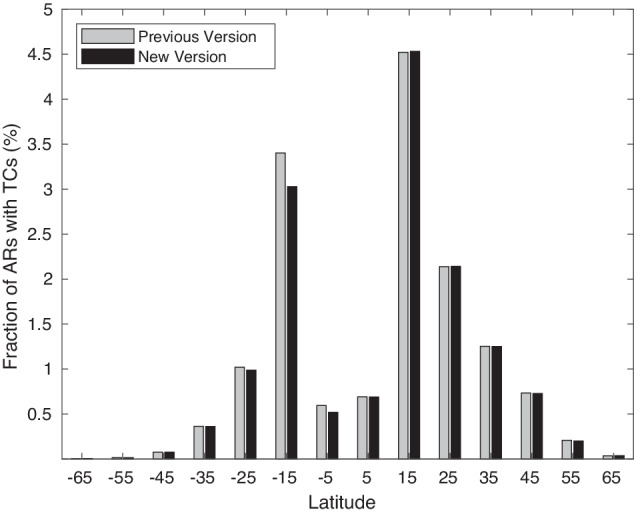
Performance of tropical cyclone filtering in two versions of the tARget algorithm. Shown is the fraction (%) of ARs with a tropical cyclone center located within the AR shape boundary as a function of latitudes. Previous version (gray) refers to version 3, and new version (black) is version 4. See Methods for details. Tropical cyclone center locations are from IBTrACS version 4^73^. Based on a coarsened (1.5° × 1.5°) version of ERA5 over 1980–2019.

In previous versions, non-AR tropical features are filtered out based on two requirements: (a) An IVT object is retained only if more than half of the pixels within the object have an IVT direction within 45° from the object-mean IVT, to filter out features related to tropical cyclones where a range of different and contrasting IVT directions can be found within the same object; (b) An IVT object is retained only if the object-mean IVT has a poleward component greater than 50 kg m^−1^ s^−1^, to filter out features related to tropical convection with primarily zonally directed IVT. In version 4, requirement (a) is replaced with the one described in step 2 above based on the standard deviation of IVT directions across individual pixels being less than 67.5° (white text on red background in Fig. 1). IVT directions are weighted by IVT magnitude and the area of individual pixels for calculating the directional standard deviation. The threshold of 67.5° is chosen such that the revised filter leads to negligible changes in climatological AR frequency (more discussion later). Compared to the previous version where pixels with an IVT direction more than 45° from the object-mean IVT are regarded as equally bad, the revised filter allows pixels with larger directional differences from the object-mean IVT to be penalized more, as is desired. In the Southern Hemisphere, the updated filter has an improved performance, where only a small or negligible fraction (<3%) of the detected ARs are potentially mis-labelled tropical cyclones (Fig. 2) – that is, it keeps tropical cyclones from a great majority of the resulting ARs in the main development region (around 15°S). In the Northern Hemisphere, the previous and new versions of the filter have nearly identical performances. In version 4, requirement (b) is replaced with the one described next in extratropical refinement. That is, even though requirement (b) is to filter features mainly found in the tropics, the refinement to it aims to improve AR detection in the extratropics. As noted earlier, all requirements in tARget are applied globally, even though some of them primarily target features found in specific regions.

#### Extratropical refinement

**Fig. 3:**
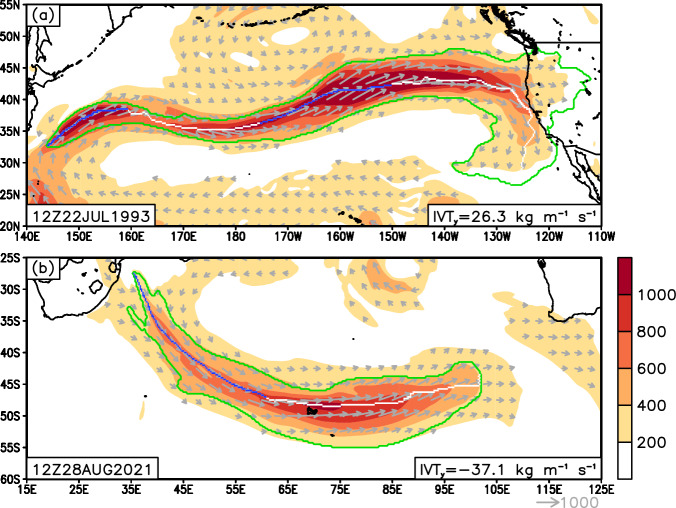
Examples of “zonal” ARs detected by tARget version 4 but undetected by the previous version. In each panel, the green contour shows the AR shape, the white/blue line shows the AR axis – blue if IVT at the given pixel has a poleward component greater than 25% of the total IVT there, and white otherwise. Shading shows IVT magnitude (kg m^−1^ s^−1^), and arrows show vector IVT where the magnitude exceeds 200 kg m^−1^ s^−1^. The mean meridional IVT within the whole AR is indicated in the bottom-right of each panel.

In the extratropics, certain ARs with a largely zonal orientation have been observed^52^, but so far have not been systematically studied. Due to their zonal orientation, the mean meridional IVT could be only weakly poleward or even equatorward for these ARs, which may therefore be inadvertently removed by requirement (b) described above. Forgoing the poleward requirement entirely is not desirable, as that would lead to numerous non-AR tropical features to be retained. By specifying the poleward requirement based on segments of the AR axis (can be located anywhere on the AR axis as long as the combined length of these segments is above 1000 km) rather than the whole AR, the refined filter described in step 3 above removes features with entirely zonal or equatorward IVT and meanwhile accommodates “zonal” ARs where a large portion of the AR may appear zonal, but still with coherent poleward IVT in certain sectors of the AR (white text on green background in Fig. 1). Examples of “zonal” ARs retained in version 4 of tARget but discarded in the previous version are shown in Fig. 3.

#### Polar refinement

**Fig. 4:**
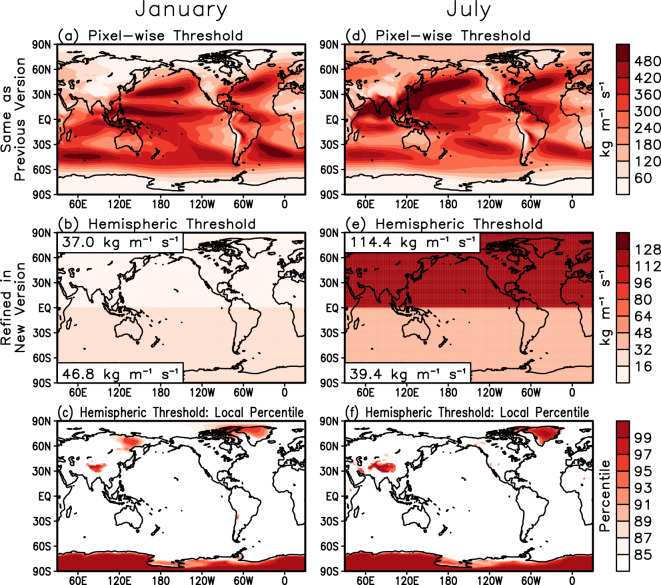
Monthly IVT thresholds used for AR detection in tARget version 4 for two example months. (top) Pixel-wise thresholds (kg m^−1^ s^−1^) based on the 85^th^ percentile of IVT. This is the same as in the previous version. (middle) Hemispheric thresholds (kg m^−1^ s^−1^) based on the area-weighted spatial 5^th^ percentile of the values shown in the top row, calculated separately for the two hemispheres. The exact values of the thresholds are indicated in the top- and bottom-left of each panel. This is a refinement in version 4 compared to the fixed lower limit of 100 kg m^−1^ s^−1^ used in the previous version. (bottom) The local temporal percentiles corresponding to the refined hemispheric thresholds shown in the middle row, for reference.

**Fig. 5:**
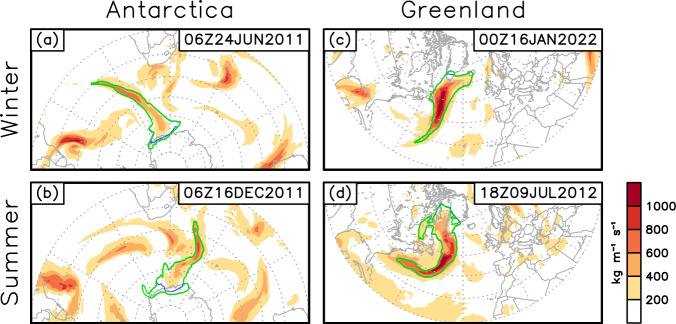
Examples of AR shapes in polar regions from two versions of the tARget algorithm. In each panel, the green contour shows the AR shape from version 4, whereas the blue contour shows the previous version (hidden by the green contour where the two versions overlap). Shading shows IVT magnitude (kg m^−1^ s^−1^).

In step 1 described above, the hemispheric IVT threshold applied on top of the pixel-wise IVT threshold effectively increases the pixel-wise threshold to higher percentiles in cold/dry regions, and serves to remove spurious features in those regions that are strong enough to pass the local 85^th^ percentile, but are nonetheless weak in terms of absolute magnitude and therefore not of practical interest. In previous versions of the tARget algorithm, a fixed lower limit of 100 kg m^−1^ s^−1^ for IVT was used to serve a similar purpose. The more nuanced specification of this lower limit in version 4, i.e., making the threshold month- and hemisphere-dependent, better aligns the tARget algorithm with polar-specific algorithms in polar regions (white text on blue background in Fig. 1). Specifying this lower limit based on the area-weighted spatial 5^th^ percentile of IVT_threshold_(x,y,m), as explained earlier, rather than an absolute value of 100 kg m^−1^ s^−1^ will also be useful in cases where the input IVT data are systematically biased, or where the input data are not IVT at all. By definition, the hemispheric IVT threshold raises the pixel-wise threshold to higher percentiles over 5% of the surface area of the corresponding hemisphere where IVT is climatologically the lowest. To illustrate, Fig. 4 shows the pixel-wise IVT threshold (top row) which remains the same as in the previous version, the hemispheric IVT threshold (middle row) which is refined from the previous version, and for reference, the local temporal percentile corresponding to the refined hemispheric IVT threshold, for two example months. In polar areas, the hemispheric threshold is equivalent to the local 95^th^ percentile or above (Fig. 4, bottom). Similarly high (98^th^) IVT percentile threshold was used in an Antarctic AR detection algorithm^53^. The absolute value of the hemispheric IVT threshold is ~50 kg m^−1^ s^−1^ in austral summer (Fig. 4, middle left), which is much smaller than the threshold of ~110 kg m^−1^ s^−1^ in boreal summer (Fig. 4, middle right), consistent with the smaller IVT threshold (50, as opposed to 150 kg m^−1^ s^−1^) used in the application of a polar-specific AR detection algorithm in Antarctica^54^ compared to the application of the same algorithm in the Arctic^55^ during their respective melt seasons. Compared to 100 kg m^−1^ s^−1^ used in the previous version, the refined hemispheric threshold has a smaller absolute value except in boreal summer (May–September), as shown in the examples in Fig. 4 (middle). As a result, the AR shapes from version 4 extend further inland in all months in Antarctica and winter in Greenland, but less inland during summers in Greenland, as shown by the examples in Fig. 5. The polar refinement of the global algorithm may better facilitate examination of the excursion of lower-latitude ARs into polar regions – a topic with increasing scientific attention due to the influence of ARs on poleward moisture and heat transport and associated extreme snow accumulation and melt events (e.g., Gorodetskaya *et al*.^56^, Neff *et al*.^57^, and many studies thereafter).

#### Comparison of AR detection result with previous version

**Fig. 6:**
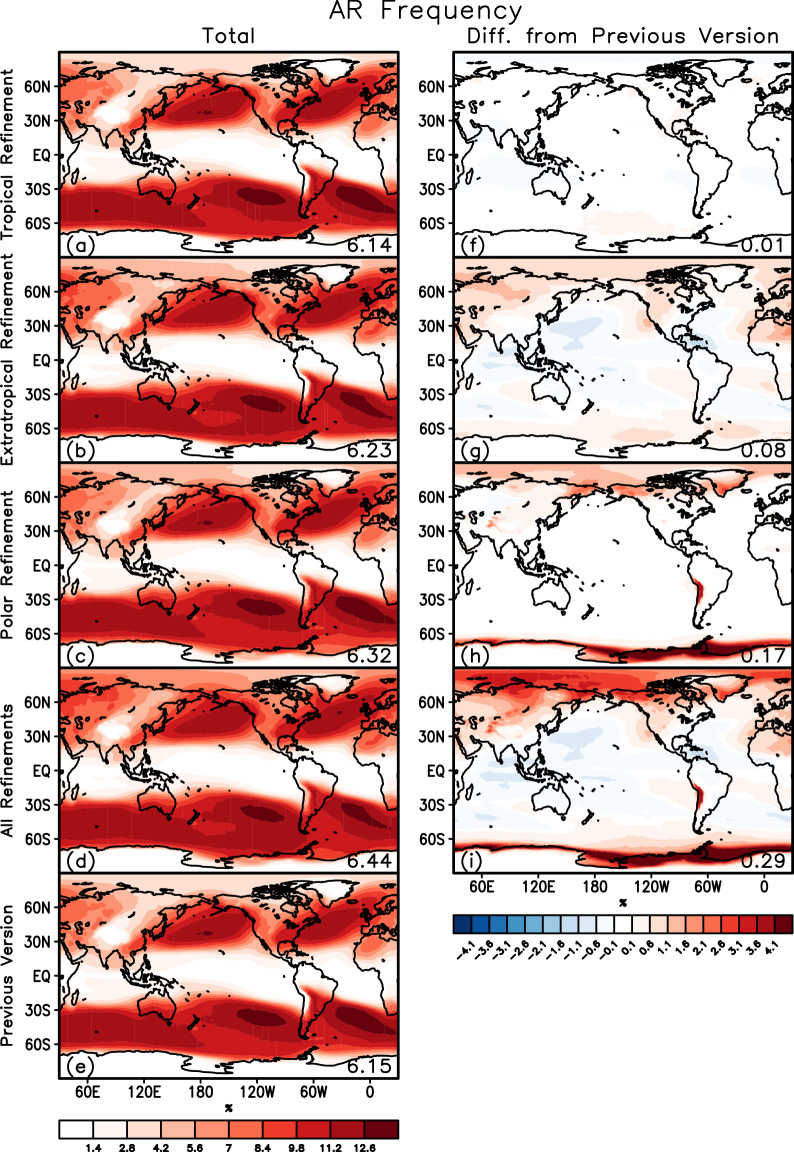
Comparison of climatological AR frequency between refined and previous versions of tARget. The left column shows the AR frequency (%) from five experiments: tropical refinement, extratropical refinement, polar refinement, all refinements combined, and the previous version. The right column shows the absolute difference in AR frequency between each of the refined versions and the previous version – note the shading threshold is 0.1% while shading intervals are 0.5%. In all panels, the number in the bottom-right corner shows the global mean. Based on a coarsened (1.5° × 1.5°) version of ERA5 over 1980–2019.

The refinements described above did not change the overall pattern of AR frequency distributions (Fig. 6). Compared to the previous version (Fig. 6e), the tropical refinement resulted in negligible changes in the climatological AR frequency (Fig. 6f). The extratropical refinement led to a global mean increase of 0.08% (based on absolute difference, not relative) in AR frequency (Fig. 6g), consistent with the detection of “zonal” ARs that went undetected in the previous version as shown by the examples in Fig. 3. Regions with increased AR frequencies include the Arctic Ocean and a number of coastal and land areas in both hemispheres. The presence of “zonal” ARs in the Arctic was noted by Mattingly *et al*.^55^, who removed the poleward IVT requirement entirely in their AR detection north of 70°N. Regions with reduced AR frequencies are also noted with the extratropical refinement. Larger changes occur with the polar refinement, especially in West Antarctica and coastal East Antarctica (Fig. 6h), consistent with the further inland extension of the AR shape boundaries as shown by the examples in Fig. 5. Interestingly, the polar refinement also resulted in notable increase in AR frequency around the Atacama Desert, the driest nonpolar desert in the world, where AR-like structure has been observed^58^. When combined, the three refinements resulted in a global mean increase of AR frequency from 6.15% to 6.44% (Fig. 6d).

## Data Records

The global AR database^59^ based on applying tARget version 4 on the ERA5 reanalysis covers the period of 1940–2023 at 6 h intervals with a horizontal resolution of 0.25° × 0.25°, and is part of the Global Atmospheric Rivers Dataverse^60–63^ (https://dataverse.ucla.edu/dataverse/ar). The algorithm was run on ERA5 data resampled to 6 h intervals to conserve computational and storage resources. The 6 h input data resolution is adequate for AR tracking using the spatial overlapping method, as discussed earlier. The sensitivity analysis in Guan and Waliser^22^ (their Fig. 5) indicated that the tARget algorithm is insensitive to the temporal resolution of the input data, but sensitive to the horizontal resolution. Therefore, the native ERA5 horizontal resolution is maintained. The output is stored in a single netCDF file with necessary metadata, and can be readily ingested into common analysis software and programming languages. Selected examples of the different types of variables are listed in Table 1. The full list of variables and related information are part of the metadata of the AR database contained in the self-descriptive netCDF file.

**Table 1:** Selected examples of different types of variables in the AR database based on tARget version 4.

	Variable Name	Dimensions	Description
Map Variable	shapemap	lon, lat, lev, time, ens	Shape
	axismap	lon, lat, lev, time, ens	Axis
	kidmap	lon, lat, lev, time, ens	Track: ID
	kstatusmap	lon, lat, lev, time, ens	Track: Status
Attribute Variable: Single Value	length	lat, lev, time, ens	Length
	width	lat, lev, time, ens	Effective Width
	width2	lat, lev, time, ens	Transect Width
	tivt	lat, lev, time, ens	Total IVT Across Transect
	ivtx & ivty	lat, lev, time, ens	Mean Zonal & Meridional IVT
	klifetime	lat, lev, time, ens	Track: Lifetime
	kivtx & kivty	lat, lev, time, ens	Track: Mean Zonal & Meridional IVT
Attribute Variable: Array	axislon & axislat	lon, lat, lev, time, ens	Longitudes & Latitudes of Smoothed Axis

For attribute variables, “lon” (if present) and “lat” dimensions are fictitious – see text for details.

Variables with names ending in “map” (hereafter, map variables) provide pixel-wise information about ARs at each time step (such as “shapemap” for maps of AR shape ID) and are on the same grid as the input IVT data, with the following five dimensions: (1) The first dimension (“lon”) represents longitude, going eastward from 0 to 359.75°; (2) The second dimension (“lat”) represents latitude, going southward from 90 to −90° following the ERA5 convention; (3) The third dimension (“lev”) is redundant, and reserved for future use; (4) The fourth dimension (“time”) gives temporal information, including the calendar used (Gregorian in this database); (5) The fifth dimension (“ens”) is redundant and reserved for future use.

Variables with names not ending in “map” (hereafter, attribute variables) provide the attributes of each AR object. The “lon” (if existing) and “lat” dimensions of these variables are fictitious and have specific interpretations as described here. AR attributes that are a single number (such as AR length, named “length”) have four dimensions, with the first dimension (“lat”) representing the index of the AR object that goes from 1 to the total number of AR objects at a given time step, and the rest 3 dimensions (“lev”, “time”, “ens”) as described earlier. AR attributes that are an array (such as longitudes and latitudes of the smoothed AR axis, named “axislon” and “axislat”) have five dimensions, with the first dimension (“lon”) representing the index of the array that goes from 1 to the total number of elements in that array, and the rest 4 dimensions (“lat”, “lev”, “time”, “ens”) as described above. The leading dimensions of the attribute variables are fictitiously named “lon” and “lat” to facilitate automatic ingestion into software that can only recognize certain dimension names due to the netCDF convention used in those software.

The data are stored with the following structure: For a given AR object at a given time step with a shape ID of *n* (indicating the *n*^th^ object at that time step), the corresponding map variable (“shapemap”) is formed by populating the number *n* to all pixels belonging to that AR object, with pixels not within any ARs assigned missing values (NaN). Other map variables are stored similarly. For AR axis (“axismap”), for example, the value *n* is populated to all pixels belonging to the AR axis, and missing values are filled in elsewhere within the AR footprint. For AR attributes that are a single number, such as AR length (“length”), the values are stored as the *n*^th^ element along the “lat” dimension in the corresponding attribute variables. For AR attributes that are an array, such as longitudes and latitudes of the smoothed AR axis (“axislon” and “axislat”), the values are stored as the *n*^th^ array along the “lat” dimension in the corresponding attribute variables.

Among all variables described above, those beginning with the letter ‘k’ are track-related (such as “klifetime” for AR lifetime). In most cases, these variables repeat the same value (such as a value of 259,200 for AR lifetime in seconds) over all time steps belonging to the specific AR life cycle. The only exceptions are the instantaneous travel velocity and track status, whose values are specific to each time step. Each AR life cycle is assigned a track ID (named “kid”) unique within the entire database. The track ID is in the format of YYYYMMDDHHID, where YYYY is year, MM is month, DD is day, HH is hour, and ID is the AR shape ID, each with the implied number of digits. By following a specific track ID in time, the entire life cycle of an AR can be followed. The status of each time step within an AR life cycle (named “kstatus”) is recorded in the format of XYZ, where X is 0 or 1 indicating whether separation occurred, Y is the same but for merger, and Z is 1 for genesis, 3 for termination, and 2 otherwise (Z is 0 for single-step AR life cycles).

## Technical Validation

### Comparison of key AR characteristics with Zhu and Newell’s early studies

**Fig. 7:**
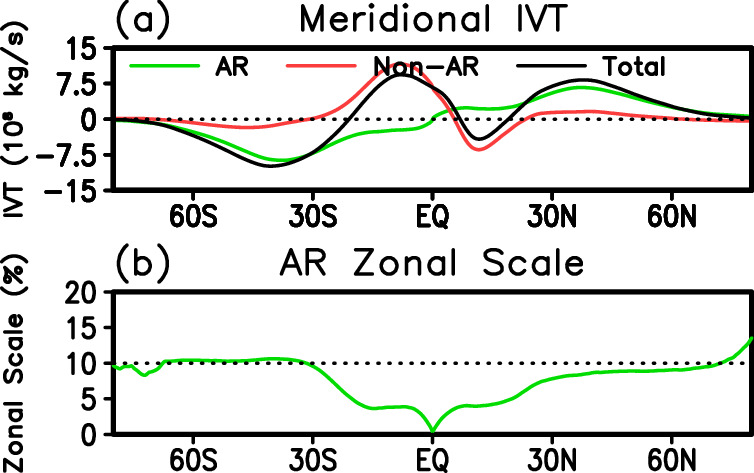
Key characteristics of ARs in ERA5. (**a**) Zonally-integrated meridional IVT (kg s^−1^) associated with ARs (green), not associated with ARs (red), and their combination (black). (**b**) Integrated AR zonal scale expressed as the fraction of the total zonal circumference at each latitude.

The first validation analysis here evaluates whether the fundamental characteristics (narrowness and magnitude of poleward moisture transport) of the ARs from the new database are consistent with the ARs studied by Zhu and Newell^1,2^, who coined the term “atmospheric river”. Based on three years of analysis data and a formulation to extract ARs without explicit consideration of AR geometry, Zhu and Newell^2^ found that ARs account for over 90% of the poleward moisture transport across the midlatitudes while occupying only about 10% of the Earth surface in those latitudes. Based on the new database, ARs account for 83% (85%) of the total meridional IVT, and 9% (10%) of the zonal circumference, between 30 and 60° in the Northern (Southern) Hemisphere (Fig. 7). The AR narrowness, including the hemispheric difference, matches Zhu and Newell^2^ (their Fig. 6) very well. In both hemispheres, the AR fractional IVT is slightly lower than indicated in Zhu and Newell^2^, which could be related to differences in the data sources, analysis periods, as well as the additional constraints on AR geometry in the tARget algorithm.

### Comparison of AR landfall dates with region-specific algorithms

The new AR database is further assessed by comparing landfall dates to three previous studies independently conducted for three regions: western North America^64^, Britain^15^, and East Antarctica^56^ (Table 2). A similar comparison with these studies was conducted in Guan and Waliser^22^ to assess the performance of the original version of the global AR database. Different variables and thresholds (3^rd^ and 4^th^ rows) were used for AR detection in these studies to suit the specific applications in those regions. Note that AR landfall dates reported in the latter two studies are only those associated with extreme hydrological events and are therefore less frequent than in the first study (5^th^ row). We compare the total number of AR landfall dates for each location reported in the three studies to the subset of those dates that have a corresponding landfall from the new database within ± 1 day in the same region, and calculate the ratio between the two counts as a measure of agreement. The 1-day offset accommodates possible timing difference in AR landfalls as a result of differences in data and methods. The percent agreement between AR landfalls from the new database and from those previous studies is 99%, 94%, and 100% for western North America, Britain, and East Antarctica, respectively (bottom row). The comparison to these independent studies and AR detection techniques suggests the new AR database has a reasonable performance across regions with different climatologies.

**Table 2:** Comparison of AR landfall dates between tARget version 4 and region-specific algorithms in three previous studies independently conducted for three regions.

Study Area	Western North America^64^	Britain^15^	East Antarctica^56^
**Period**	1997–2014, November–March	1997–2010, October–March	2009–2012, All Months
**Variable for AR Detection**	IWV from SSM/I and SSMIS Retrievals	900-hPa Specific Humidity from Twentieth Century Reanalysis Project	IWV from ERA-Interim Reanalysis
**Intensity Criterion for AR Detection**	20 mm	5 g/kg	IWVsat,mean + 0.2(IWVsat,max − IWVsat,mean), where IWVsat is saturation IWV, and mean/max refers to zonal mean/maximum
**Number of Landfall Dates**	347	18 (high-impact events only)	13 (high-impact events only)
**Number of Landfall Dates in Agreement with Current Database**	343	17	13
**Percent Agreement**	99%	94%	100%

The period listed is the overlapping period between the current database and each of the three studies. All three studies used similar geometric criteria for AR detection (approximately >2000 km long and <1000 km wide). Number of dates in agreement is based on a ± 1 day search window. Percent agreement is based on the ratio between the number of dates in agreement and the total number of landfall dates reported in the respective studies.

### Comparison of AR width and total IVT with dropsonde observations

In the above, we have evaluated the narrowness of ARs represented in the new database based on an aggregate metric, that is, the fraction of total zonal circumference accounted for by all ARs at a given latitude. Here, we further evaluate the narrowness of ARs by explicitly comparing the width of individual ARs to dropsonde observations, and while doing so, evaluate the total IVT (TIVT) across the AR width – a useful AR metric implicated in Zhu and Newell^2^ and formally defined in Ralph *et al*.^65^. The AR width (“width2”) evaluated here represents the width of the transect that goes through the AR centroid in a direction perpendicular to the mean AR IVT. This is different from the other metric of AR width (“width”) also recorded in the database, which is simply the ratio between the area of an AR and its length, that is, the effective width. The centroid-based AR width, rather than the effective AR width, is evaluated here because it is more consistent with the way ARs are observed by dropsondes, i.e., along transects across the ARs that attempt to sample the center part of ARs^65^. Defined as the integration of the IVT component perpendicular to the transect that defines the AR width, TIVT (“tivt”) is analogous to the streamflow in a terrestrial river. Both the AR width and TIVT are part of the AR attributes recorded in the tARget database.

**Fig. 8:**
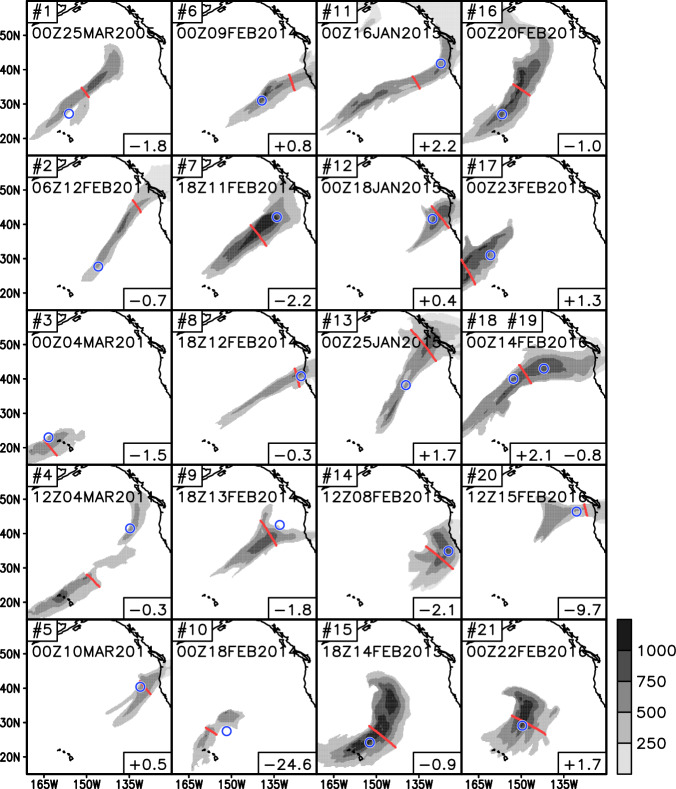
The 20 ERA5 reanalysis ARs corresponding to 21 ARs transected by dropsondes. The numbering of the transect in the top-left of each panel is as in Ralph *et al*.^65^. Shown is the 6-h time step – also indicated in the top-left of each panel – of the reanalysis AR (shading: IVT magnitude, kg m^−1^ s^−1^; red: transect) closest in time to the mid-point of the dropsonde transect (blue). Two of the 21 dropsonde transects (#18 and #19) correspond to the same 6-h step of the reanalysis AR (3rd panel in the last column). Indicated in the bottom-right of each panel is the time difference (h) between the closest 6-h step of the reanalysis and the mid-point of the dropsonde transect (as a nominal observational time), where positive/negative signs represent that the reanalysis lags/leads the observation.

The evaluation here follows similar procedures used in Guan *et al*.^23^ to evaluate the second version of the AR database. Based on the time and location of the 21 dropsonde-observed AR transects reported in Ralph *et al*.^65^, we identified 20 corresponding ARs from the ERA5 reanalysis based on searching the 6-h step of ERA5 that has an AR in the vicinity of the dropsonde transect and closest in time to the mid-point of the transect (Fig. 8) – two of the dropsonde transects (#18 and #19) were close enough in time to correspond to the same reanalysis time step. Despite the limited number, a variety of ARs are sampled, such as landfalling versus offshore, subtropical versus midlatitude, and long-and-narrow versus less well elongated. Nineteen out of the 21 dropsonde transects have a matching reanalysis AR within ± 3 hours, that is, within the temporal resolution of the AR database. The other two dropsonde transects have a matching reanalysis AR 24.6 and 9.7 h earlier, respectively. In 11 cases, the dropsonde and reanalysis transects are spatially close to each other. The location difference in other cases is partly attributable to difference in data and methods used for AR detection, but also the timing difference between dropsonde and reanalysis, during which an AR can propagate in space and/or change in structure.

**Fig. 9:**
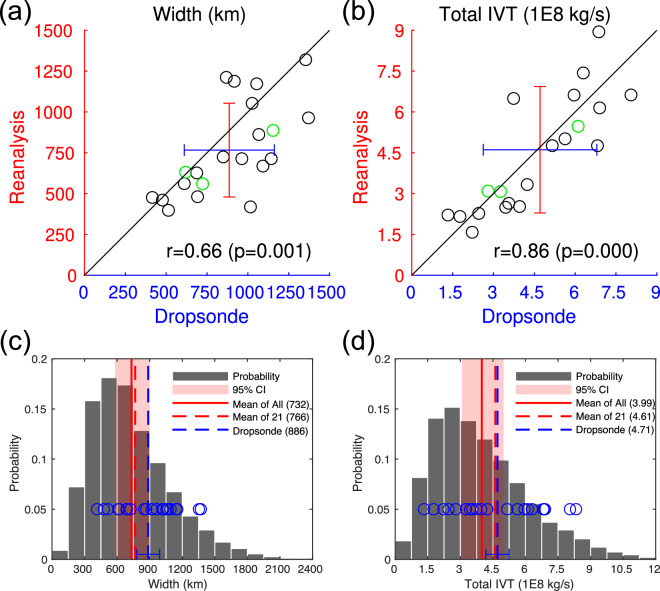
Comparison of ARs between dropsonde observations and the ERA5 reanalysis. (**a**) AR width (km). See Fig. 8 for the time and location of each transect. The three cases with the best space-time coincidence between dropsonde and reanalysis AR transects (#5, #8, and #12 in Fig. 8) are shown in green. Blue and red error bars indicate one standard deviation below/above the mean (where the two error bars cross). (**b**) As (a) but for TIVT (10^8^ kg s^−1^). (**c**) Histogram of AR width (km) based on all reanalysis ARs detected over the northeastern Pacific (AR centroids within 163.4–124.6°W, 23–46.4°N) during 15 January to 25 March of 1940–2023 (gray bars). Also shown are the mean AR width (km) based on all reanalysis ARs (red solid), the subset that corresponds to the 21 dropsonde transects (red dashed), and the 21 dropsonde transects themselves (blue dashed for the mean, and blue circles for individual transects). The mean AR width is also indicated in the figure legend for each set. Red shading indicates the 95% confidence interval of the mean reanalysis AR width for a random 21-member sample drawn from the pool of all reanalysis ARs based on 10,000 iterations. The error bar centered on the blue dashed line indicates the 95% confidence interval of the difference between the blue and red dashed lines based on a two-tailed, paired *t*-test. (**d**) As (c) but for TIVT (10^8^ kg s^−1^).

We now quantitatively compare AR width and TIVT between dropsonde observations and the ERA5 reanalysis. In Fig. 9, each circle represents one pair of dropsonde and reanalysis transects (see Fig. 8, blue and red). Note that while the reanalysis AR width is based on the IVT 85^th^ percentile, the dropsonde AR width is based on a fixed IVT threshold of 250 kg m^−1^ s^−1^. The two sets of AR widths are correlated with a correlation coefficient of 0.66 (*p* = 0.001) (Fig. 9a). The blue and red error bars indicate one standard deviation below/above the mean (where the two error bars cross). The reanalysis- and dropsonde-based results have similar standard deviations, but reanalysis exhibits a bias of −13.5% in mean AR width (see also Fig. 9c). A comparison to the similar analysis in Guan *et al*.^23^ but based on the coarser-resolution predecessor of ERA5, where a bias of −2% is found, suggests the width of detected ARs is somewhat sensitive to the horizontal resolution of the input data, with higher resolution leading to narrower ARs – consistent with the sensitivity test in Guan and Waliser^22^ (their Fig. 5l). The agreement between dropsondes and reanalysis is better for TIVT, with a stronger correlation coefficient of 0.86 (*p* < 0.001), and largely matching means and standard deviations. The better agreement for TIVT compared to AR width regardless of the input data for AR detection (comparing to Guan *et al*.^23^) suggests TIVT is a more robust AR metric. This is attributable to the integration being dominated by strong IVT values toward the AR center and therefore insensitive to small errors in the detected AR width.

The three cases with the best space-time coincidence between dropsonde and reanalysis AR transects (green circles in Fig. 9a,b; see #5, #8, and #12 in Fig. 8) are among the cases, but not the only cases, with the best agreement in AR width and TIVT (i.e., circles located close to the 1:1 line). This is expected as the degree of agreement is affected, but not solely determined, by the space-time coincidence, because the dropsonde observation and the reanalysis each independently aims to transect the most influential part of the AR based on where they respectively identify that part to be. As such, the object-level error statistics discussed above for AR width and TIVT – accommodating reasonable mismatch in time and space between objects – should not be directly translated to reanalysis errors in the regular sense.

In the above, we evaluated the agreement in AR statistics between ARs matched in dropsonde observations and the reanalysis. Next, we further assess if AR statistics based on the 21 dropsonde-observed ARs are consistent with a random sample from the full population of ARs represented in the reanalysis. To facilitate comparisons, we extracted ARs detected in the northeastern Pacific (AR centroids within 163.4–124.6°W, 23–46.4°N) during 15 January to 25 March of 1940–2023. The domain and the combination of days selected are just enough to encompass the location and time of the 21 dropsonde-observed ARs. Both AR width and TIVT are characteristic of a log-normal distribution with a longer right tail based on the total of >12,000 reanalysis ARs selected (Fig. 9c,d, gray). As already discussed above, mean AR width in the reanalysis exhibits a negative bias, while mean AR TIVT well matches dropsonde observations (Fig. 9c,d, comparing red and blue dashed lines). Sampling variations in mean AR width and TIVT are obtained based on randomly selecting 21 ARs from the pool of all reanalysis ARs, repeating for 10,000 times, and finding the 2.5^th^ and 97.5^th^ percentiles of the resulting empirical distributions (Fig. 9c,d, red shading). For AR width (Fig. 9c), the dropsonde-mean (blue dashed) is within the sampling variations of the reanalysis but close to the upper bound (right edge of red shading). For TIVT (Fig. 9d), the dropsonde-mean is well within the sampling variations of the reanalysis. The result supports that the 21 dropsonde-observed ARs are consistent with a random sample of the same size from the full population of northeastern Pacific ARs in terms of key AR characteristics such as width and TIVT.

### Comparison of AR durations with ground observations from an AR observatory

**Fig. 10:**
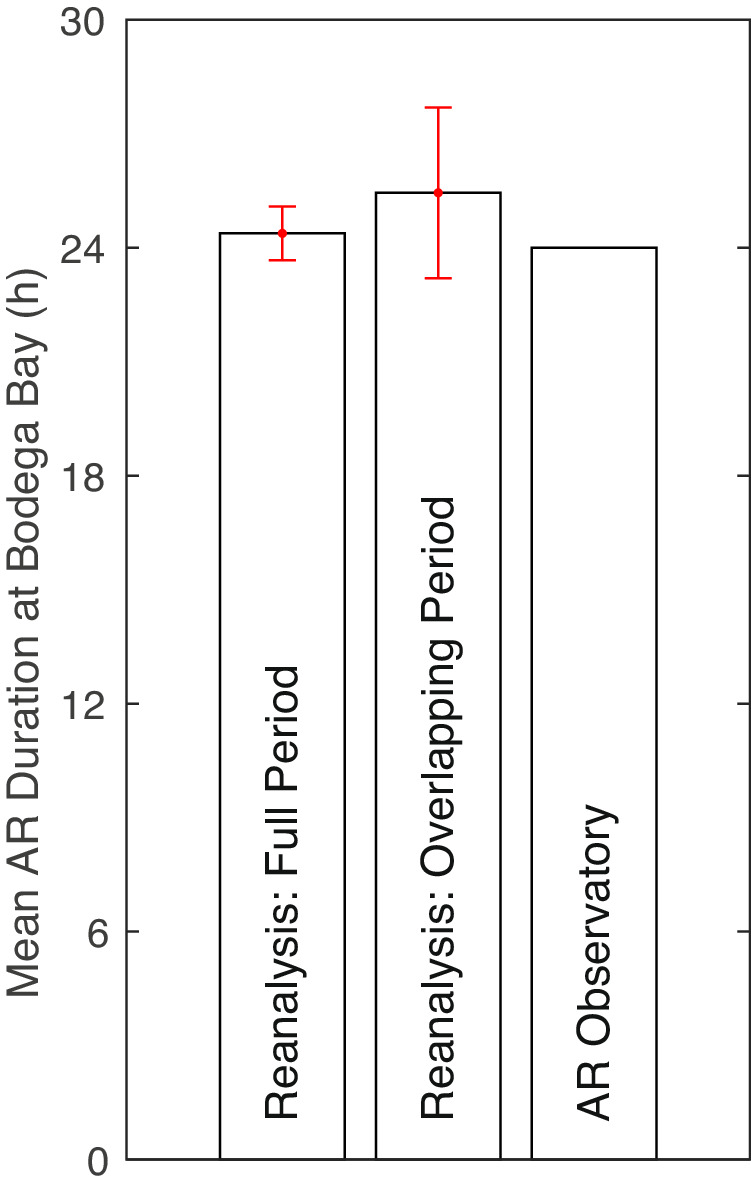
Mean AR duration (h) based on three sets of ARs at Bodega Bay, California. The left bar is based on ARs over the full period of the ERA5 reanalysis for the grid cell that contains the observational site, the center bar is the same except based on the overlapping period with observations, and the right bar is based on observations from an AR observatory at Bodega Bay, California during 2004–2015^66^. The red error bars indicate the 95% confidence interval for the reanalysis ARs.

Finally, we evaluate mean duration of the reanalysis ARs against ground observations. AR event duration is based on counting the number of hours AR conditions are continuously met at a given location – that is, a Eulerian perspective. Using hourly observations from an AR observatory at Bodega Bay, California during 2004–2015, Ralph *et al*.^66^ defined AR events to be when (1) the integrated water vapor (IWV) exceeds 20 mm, (2) the IWV flux exceeds 200 mm m s^−1^, and (3) the two conditions are simultaneously met for at least 12 consecutive hours. The mean duration for a total of 114 AR events thus defined is 24 h (Fig. 10, right bar).

Based on reanalysis ARs detected at the grid cell containing the Bodega Bay station over the entire period of 1940–2023, the mean AR duration is 24 h – counting only those ARs with durations of at least 12 h, to match the threshold used in the observational reference. The mean AR duration based on the overlapping period with the observations is 25 h. In either case, the 95% confidence interval encompasses the mean observed value, suggesting that the reanalysis ARs are non-biased relative to the observed in terms of event duration – an AR metric closely related to AR impacts^8,33,67,68^.

## Usage Notes

Using the AR database, a variety of AR statistics can be derived. Only a few examples are given here. Climatological AR frequency at a given grid cell can be calculated as the ratio between the number of time steps when the AR shape ID (variable “shapemap”) is non-NaN and the total number of time steps over the analysis period. AR IVT at each grid cell can be obtained by masking the raw IVT based on whether the AR shape ID is NaN (note the variables “ivtx” and “ivty” included in the database are for object-mean IVT, not IVT at individual grid cells). Using the masked IVT, climatological AR IVT at each grid cell can then be obtained by averaging over non-NaN values. AR genesis and termination frequencies at each grid cell can be obtained by counting the number of time steps at which the last digit of track status (variable “kstatusmap”) is 1 (for genesis) or 3 (for termination). AR track frequency at each grid cell can be obtained by counting the number of unique track IDs (variable “kidmap”). A mix of GrADS and MATLAB codes for calculating a suite of 17 AR metrics are available^69^. In addition to enabling the analysis of long-term AR variability and trends, the database is expected to be extended in time annually to facilitate the analysis of the latest events of interest.

## Data Availability

The AR detection algorithm, tARget version 4^70^, is available via the Global Atmospheric Rivers Dataverse (https://dataverse.ucla.edu/dataverse/ar).
